# Synchronous Remote Teaching for Neurological Examination Training in Fifth-Year Medical Students: Quasi-Experimental Study

**DOI:** 10.2196/77034

**Published:** 2026-07-15

**Authors:** Chih-Shan Huang, Kung-Pei Tang, Ming-Shun Wu

**Affiliations:** 1 Department of Neurology, Wan Fang Hospital, Taipei Medical University, Taipei, Taiwan; 2 Department of Neurology, School of Medicine, College of Medicine, Taipei Medical University, Taipei, Taiwan; 3 Taipei Neuroscience Institute, Taipei Medical University, Taipei, Taiwan; 4 Department of Early Childhood and Family Education, College of Education, National Taipei University of Education, Taipei, Taiwan; 5 Division of Gastroenterology, Department of Internal Medicine, Wan Fang Hospital, Taipei Medical University, No 111, Sec 3, Xinglong Rd, Wenshan Dist, Taipei, 11696, Taiwan, 886 2 2930 7930 ext 8103; 6 Division of Gastroenterology and Hepatology, Department of Internal Medicine, School of Medicine, College of Medicine, Taipei Medical University, Taipei, Taiwan

**Keywords:** synchronous remote teaching, neurological examination, medical education, quasi-experimental study, self-regulated learning, technology acceptance

## Abstract

**Background:**

The use of remote teaching in medical education has increased since the COVID-19 pandemic. However, the effectiveness of synchronous remote teaching for specific psychomotor components of the neurological examination, such as tendon reflex assessment, remains underexplored.

**Objective:**

This study aimed to evaluate the short-term effectiveness of synchronous remote teaching compared with traditional in-person teaching for training medical students in tendon reflex examination skills and related neurological examination knowledge.

**Methods:**

This quasi-experimental study enrolled 110 fifth-year medical students between August 2022 and April 2024, who were assigned to either synchronous remote teaching (n=46) or in-person teaching (n=64). The participants completed pre- and postcourse knowledge tests and an objective structured clinical examination (OSCE) assessing tendon reflex examination skills. Motivational beliefs related to self-regulated learning and technology acceptance were measured at baseline. Group differences were analyzed using *t* tests, Mann-Whitney *U* tests, and correlational analyses.

**Results:**

Posttest knowledge scores were higher in the synchronous remote teaching group than in the in-person group (mean difference 0.86, 95% CI 0.21‐1.51; Cohen *d*=0.50; *P*=.01), indicating a modest effect size. Both groups demonstrated substantial improvements in tendon reflex OSCE performance; however, the between-group difference in OSCE score improvement was not statistically significant (mean difference −3.64; 95% CI −9.36 to 2.08; Cohen *d*=−0.26; *P*=.21). Within the synchronous remote teaching group, higher motivational beliefs were positively correlated with OSCE score gains (Pearson *r*=0.34; *P*=.04; n=46), suggesting a potential role of self-regulated learning–related motivational processes. These exploratory findings should be interpreted with caution.

**Conclusions:**

Under the conditions of this study, synchronous remote teaching was associated with modest improvements in knowledge outcomes and with OSCE-assessed tendon reflex examination skill gains that were comparable to those of traditional in-person teaching, without statistically significant between-group differences in psychomotor performance. These findings do not establish durable skill acquisition and should not be interpreted as evidence of equivalence or superiority of synchronous remote teaching over in-person instruction. Unlike many prior remote medical education studies that primarily focused on asynchronous learning or self-reported outcomes, this study incorporated synchronous interactive instruction with objective OSCE-based psychomotor assessment. These findings contribute to the emerging literature on synchronous remote clinical skills education, although future randomized and longitudinal studies are needed to evaluate long-term skill retention and broader applicability.

## Introduction

The COVID-19 pandemic has accelerated the adoption of remote teaching in medical education [1-3]. Compared with traditional face-to-face instruction, remote teaching offers flexibility by reducing geographical and physical constraints [4]. It is commonly categorized into asynchronous and synchronous formats [5]. While asynchronous learning allows self-paced study, synchronous teaching enables real-time interaction and feedback, which may be particularly important for training psychomotor clinical skills [6]. Psychomotor skills require not only knowledge acquisition but also supervised practice, immediate feedback, and performance correction. These requirements present challenges for remote teaching environments, where the absence of physical presence may limit instructors’ ability to observe and guide learners effectively [7]. Neurological examination is a core clinical competency used to localize lesions and guide diagnostic reasoning [8]. Many components, such as tendon reflex assessment, are psychomotor skills that require precise technique, appropriate force application, and timing [9]. These skills are therefore highly dependent on direct observation, guided practice, and feedback, making them particularly relevant for evaluating the effectiveness of synchronous remote teaching [10].

Recent studies have further demonstrated that online and synchronous learning modalities can effectively support medical education, particularly when interactive elements are incorporated [11-13]. Synchronous remote teaching may address some limitations of asynchronous formats by enabling real-time supervision and interaction [14]. Prior educational studies have suggested that synchronous online learning environments may enhance learner engagement and cognitive participation through immediate interaction and feedback mechanisms [15]. With video-based platforms, instructors can observe learners’ performance, provide immediate feedback, and correct errors during skill execution [16-18]. However, whether such real-time interaction can translate into objectively measurable improvements in clinical performance remains unclear. From a theoretical perspective, learner engagement in synchronous remote environments may be understood through the technology acceptance model (TAM) [19], which emphasizes perceived usefulness and perceived ease of use as key determinants of technology adoption [20]. Recent bibliometric analyses further suggest that TAM remains a dominant theoretical framework for understanding learner engagement and behavioral intention in contemporary online education environments, particularly in technology-mediated interactive learning settings [21]. In addition, self-regulated learning (SRL) theory highlights the role of learners’ active monitoring, goal setting, and self-efficacy in skill acquisition [22]. Core SRL components relevant to psychomotor skill acquisition include goal setting, self-monitoring of performance against criteria, and self-efficacy, which influences effort, persistence, and responsiveness to feedback [23]. These frameworks suggest that both technological factors and learner-related processes may influence learning outcomes in remote clinical skills training.

Despite these theoretical considerations, empirical evidence regarding the effectiveness of synchronous online teaching for psychomotor clinical skills, particularly in relation to objectively assessed neurological examination performance, remains limited and inconsistent [24]. Prior studies have largely focused on asynchronous learning or knowledge-based outcomes, with relatively few directly comparing synchronous remote teaching with traditional in-person instruction using standardized performance-based assessments such as objective structured clinical examinations (OSCEs) [25-28]. Evidence specifically addressing discrete psychomotor tasks, such as tendon reflex examination, remains particularly scarce. To address this gap, we conducted a quasi-experimental study to compare the short-term effectiveness of synchronous remote teaching and traditional in-person teaching among fifth-year medical students. The primary outcome was OSCE-assessed tendon reflex examination performance, and the secondary outcome was neurological examination knowledge. We hypothesized that synchronous remote teaching would achieve similar short-term improvements in both OSCE performance and knowledge compared with in-person teaching.

## Methods

### Study Design

This study used a neurological examination course integrated into the physical examination curriculum at a medical center affiliated with a university in Taipei. This study used a nonrandomized, open-label, active-control, parallel-group design with within-participant repeated measurements (pretest-posttest). Data were collected at the following two predefined time points: baseline before the course (T0) and immediately after course completion (T1), with motivation and technology acceptance measured once at baseline.

### Setting

The study was conducted within the neurological examination curriculum for fifth-year medical students at a university-affiliated medical center in Taipei, Taiwan. Participant recruitment and data collection were conducted from August 29, 2022, to April 22, 2024.

### Participant Recruitment

Fifth-year medical students (clerkship 1) rotating through internal medicine and neurology were recruited during the study period. Because the assessments and questionnaires were embedded within the formal curriculum, participation was considered quasi-voluntary. The students participated in the regular basic clinical skills neurological examination course, with each class including approximately 8‐10 students. Because different clerkship blocks varied in size and instructional modality across the study period, the number of participants in each teaching group was not identical. Students were informed that questionnaire completion was optional and that declining participation would not affect course grades, clinical evaluations, or academic standing. Survey responses were anonymized and deidentified before analysis.

### Inclusion and Exclusion Criteria

Inclusion criteria included fifth-year medical students enrolled in the neurological examination course during the study period. Exclusion criteria included inability to complete the pre- and posttests, failure to complete course procedures, or missing any major component of the study assessments.

### Sample Size and Group Allocation

This study was designed as a pragmatic educational evaluation embedded within the existing clerkship curriculum. Therefore, a formal a priori sample size calculation based on a prespecified effect size, α level, and statistical power was not performed. Participant recruitment and group assignment were determined by cohort availability and the preexisting teaching schedule during the study period, rather than by an optimal allocation strategy (eg, Neyman allocation). As a result, group sizes were unequal between the synchronous remote teaching and the in-person teaching groups.

Specifically, the control group consisted of students who received standard in-person teaching as part of the routine curriculum. The experimental group comprised students from the same academic year who were additionally invited to participate in synchronous remote teaching. Both groups received comparable instructional content and opportunities for practice and feedback, differing only in the mode of delivery. As participation in the remote group was based on additional invitation and voluntary agreement, group allocation reflects convenience sampling without randomization; therefore, selection bias and baseline imbalance cannot be fully excluded.

### Teaching Intervention and Instructional Procedures

The experimental group used the Remote Clinical Presence System (RCPS), an enhanced telepresence setup developed for real-time remote clinical teaching, with permission granted for its use in this study. RCPS was designed to support synchronous remote teaching in clinical settings by preserving real-time interaction between instructors and learners [29]. Google Chrome was used as the web interface, and Google Meet was used for audiovisual communication.

RCPS enabled bidirectional, real-time audiovisual interaction, allowing students to observe neurological examination demonstrations and receive immediate feedback while performing examination tasks remotely. To facilitate transmission of fine motor actions and examination techniques—such as tendon localization, patient positioning, and reflex hammer handling—the camera setup provided a stable and close-up view of the examination field. Viewing angles could be adjusted during teaching sessions based on instructional needs, and instructors were able to request repositioning of the camera or repeat demonstrations to ensure adequate visualization of key procedural steps.

Before each teaching session, audiovisual connectivity was checked to ensure stable transmission. In the event of transient technical interruptions, instruction resumed after reconnection, and critical examination steps were repeated as needed to maintain instructional continuity. This instructional approach is consistent with prior reports on enhanced telepresence systems and synchronous digital platforms, which emphasize real-time interaction, demonstration, and feedback as essential elements for skill-based clinical education [29-32].

This study used a quasi-experimental design of “nonequivalent experimental group, control group with pre- and posttests” (nonrandomized controlled trial). The control group consisted of students who received in-person teaching in the classroom. These students observed the instructor’s interactions with standardized patients, demonstrations of neurological examination procedures, and the results. The instructor interacted with the students, observed how they performed the procedures, and provided feedback.

The experimental group participated in synchronous remote video learning using RCPS. These students also observed interactions, demonstrations, and results via video. The instructor interacted with them through video conferencing, observed how they performed the procedures, and provided feedback.

The neurological examination course was conducted over 2 weeks, with each session lasting about 60 minutes, using 2 standardized patient scenarios. One scenario covered the motor and sensory systems, while the other covered the cranial nerves and cerebellar systems. The goal was to train students to focus on appropriate examination strategies based on patient symptoms. The instructor demonstrated techniques, interacted with students, pointed out common errors, and allowed time for students to practice performing the neurological examination. The instructor monitored both in-person and remote students, offering real-time feedback.

### Measures and Covariates

The participants completed assessments electronically via a QR code–linked Google questionnaire at two time points (T0 baseline and T1 immediately after the course). At T0 (pretest), they completed background questionnaires, knowledge tests, self-reported confidence items, and baseline measures of motivation and technology acceptance using 5-point Likert scales based on the Motivated Strategies for Learning Questionnaire (MSLQ) [33] and the TAM [34]. At T1 (posttest), they repeated the knowledge and confidence assessments. In this study, self-reported confidence referred to their perceived comfort and readiness to perform the examination, rather than an objectively observed psychomotor skill. Self-reported confidence was measured using a 5-point Likert scale (1=not confident at all; 5=very confident), administered at both T0 and T1.

The course evaluation questionnaire and knowledge tests were reviewed by a panel of neurology educators to ensure content relevance, clarity, and alignment with course objectives. Revisions were made based on expert consensus before administration. The knowledge assessment was designed to cover key neurological domains and cognitive processes relevant to the course and was divided into pre- and posttests with comparable content and difficulty [35].

### OSCE Assessment Procedures

The participants also underwent an OSCE administered at both T0 (pretest) and T1 (posttest) using the same standardized checklist and scoring rubric. They conducted tendon reflex assessments on standardized patients at the following five specific sites: biceps brachii, brachioradialis, triceps brachii, knee jerk, and ankle jerk. The evaluation criteria for this test included the following components:

1. Instruction: explaining the examination procedure to the patient.
2. Preparation: assisting the patient to adopt the correct position, identifying the correct tendon location, and ensuring the examined area was relaxed.
3. Technique: holding the reflex hammer correctly and striking the precise tendon location.
4. Response: eliciting a tendon reflex and comparing the response on both sides.

Neurologists, trained as examiners, observed the students and graded their performance using a standardized checklist and scoring rubric. Examiners were blinded to participants’ group allocation to minimize assessment bias and were not involved in the instructional sessions. Before the study, all examiners participated in a consensus meeting to standardize the interpretation of scoring criteria and ensure consistency in evaluation. Interrater reliability was assessed before study initiation, with 2 examiners independently rating 5 students; the resulting Spearman correlation coefficient was 0.70, indicating acceptable agreement. In this study, the only directly observed psychomotor skill assessment was the tendon reflex examination OSCE, which served as a focused proxy for selected neurological examination skills. This focused approach was chosen to ensure feasibility and consistency within the course context.

### Statistical Analysis

Motivation and technology acceptance were treated as baseline learner characteristics, whereas knowledge scores, self-reported confidence, and OSCE performance were analyzed as repeated-measures outcomes. Before inferential analyses, normality of continuous variables and their change scores (T1-T0) was assessed using the Shapiro-Wilk test, supplemented by visual inspection of histograms and Q-Q plots.

Because outcomes were measured at two time points (T0 and T1), within-group pre-post changes were examined using paired-sample *t* tests when normality assumptions were met; otherwise, Wilcoxon signed-rank tests were applied. Between-group differences in change scores were examined using independent-sample *t* tests or Mann-Whitney *U* tests, as appropriate based on distributional assumptions.

Motivation and technology acceptance were further examined using Pearson correlation analyses to assess their associations with improvements in knowledge and OSCE performance.

To address baseline imbalances inherent to the nonrandomized design, regression-based, covariate-adjusted analyses were conducted using an analysis of covariance (ANCOVA) framework, with posttest outcomes specified as dependent variables, teaching modality as the fixed factor, and baseline performance and prior neurology rotation experience included as covariates. To assess whether prior neurology rotation experience moderated the effect of teaching modality, a teaching modality × prior neurology rotation interaction term was additionally included in the ANCOVA models, and *P* values for interaction were reported. Adjusted estimates with 95% CIs were reported to aid interpretation of the magnitude and precision of observed effects.

Subgroup analyses by tendon reflex examination steps, anatomical sites, and prior neurology rotation experience, as well as correlation analyses, were conducted as exploratory, hypothesis-generating analyses. Given their exploratory nature, no formal correction for multiple comparisons (eg, Bonferroni adjustment) was applied; therefore, the potential risk of type I error inflation was acknowledged, and findings were interpreted cautiously with emphasis on effect sizes and CIs.

Given the low proportion of missing data, complete-case analysis was performed, and no imputation procedures were applied. Study data were organized using Microsoft Excel (Microsoft Corp) and analyzed with SPSS (version 22; IBM Inc). All statistical tests were 2-tailed, with a 95% CI, and statistical significance was set at *P*<.05.

### Ethical Considerations

This study was approved by the Taipei Medical University Joint Institutional Review Board (approval N202201046). Written informed consent was obtained from all participants before study participation. Participation in questionnaire completion was voluntary. Students were informed that declining participation or skipping questionnaire items would not affect course grades, clinical evaluations, or academic standing. Survey responses and study data were anonymized and deidentified before analysis. All participants received a convenience store gift voucher valued at 100 New Taiwan Dollars (approximately US $3.17) as compensation for study participation. No identifiable participant images or personal information were included in the paper.

## Results

The course evaluation questionnaire and knowledge assessment demonstrated acceptable content coverage and internal consistency for the intended educational context.

**Figure 1.**
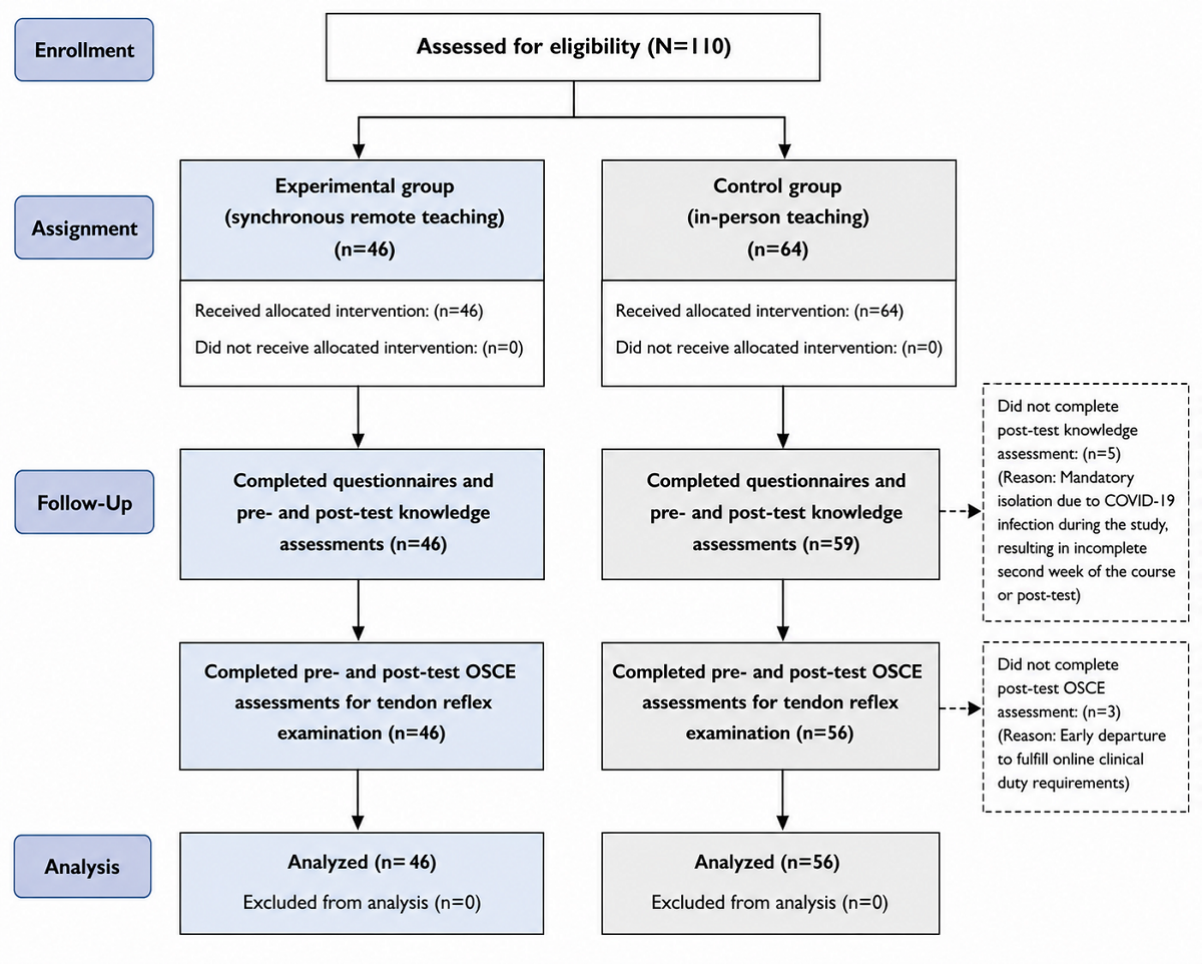
Participant flowchart of the quasi-experimental study comparing synchronous remote teaching and traditional in-person teaching for neurological examination training among fifth-year medical students at a university-affiliated medical center in Taipei, Taiwan (August 2022-April 2024). OSCE: objective structured clinical examination.

This study recruited a total of 110 participants, of whom 46 (21 female, 45.7%; mean age 22.9, SD 1.3 years) were assigned to the experimental group (synchronous remote teaching). The control group (in-person teaching) included 64 participants (19 female, 29.7%; mean age 23.2, SD 1.9 years). No significant differences were found between the groups in terms of sex (*P*=.11) or mean age (*P*=.31). All participants completed a course evaluation survey, a pretest on neurological examination knowledge, and a pretest for the OSCE on tendon reflexes. In the control group, 5 participants did not complete the second week of the course or posttest because they were under mandatory isolation following COVID-19 infection during the study period, leaving 59 participants for the posttest. In addition, 3 participants were unable to complete the OSCE posttest because they had to leave early to fulfill online clinical duty requirements, reducing the final sample size to 56. The recruitment process is shown in Figure 1.

**Figure 2.**
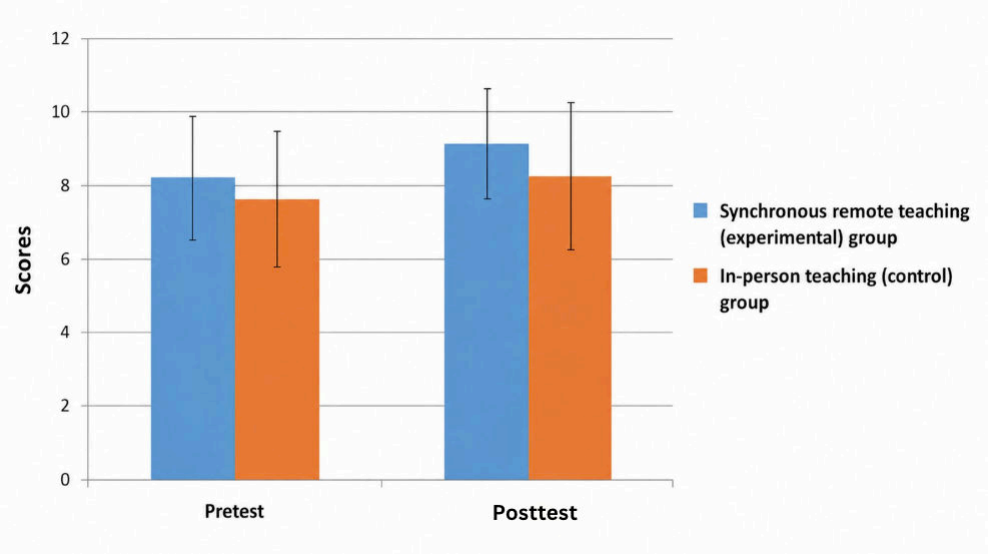
Comparison of pretest and posttest scores for the neurological examination knowledge assessment between the synchronous remote teaching (experimental) group and the in-person teaching (control) group.

Of the 110 participants, 105 completed both the pre- and posttests for the knowledge assessment and course evaluations. The Cronbach α for the MSLQ was 0.882, indicating good reliability, while the overall Cronbach α for the TAM was 0.937, indicating excellent reliability. The knowledge test items demonstrated a moderate difficulty index of 0.64 and an acceptable discrimination index of 0.23, supporting their suitability for educational assessment. Within-group analyses showed that the participants in the experimental group (n=46) had a significant increase in knowledge scores (mean 9.15, SD 1.42 vs mean 8.25, SD 1.63; *P*<.001), whereas the control group (n=59) had a smaller and nonsignificant improvement (mean 8.29, SD 1.95 vs mean 7.69, SD 1.79; *P*=.08). Between-group comparison indicated that the posttest knowledge scores were higher in the experimental group than in the control group (mean 9.15, SD 1.42 vs mean 8.29, SD 1.95; *P*=.01; Cohen *d*=0.50), corresponding to a moderate effect size. However, the absolute difference was less than 1 point on a 13-point scale, suggesting that the magnitude of this difference may be of limited practical educational significance. Notably, there was a baseline imbalance in prior neurology rotation experience between groups (42/46, 91.3% vs 43/59, 72.9%; *P*=.02), which may have partially contributed to the observed between-group difference in posttest scores. Importantly, no significant between-group differences were observed in pretest scores or in the magnitude of improvement from pre- to posttest, as shown in Table 1 and Figure 2, indicating that the overall learning gains were comparable between the 2 teaching modalities.

**Table 1.** Comparison of neurological examination knowledge between the experimental and control groups.

Characteristic	Experimental group (n=46)	Control group (n=59)	*t* test (*df*)	*P* value
Female, n (%)	21 (45.7%)	16 (27.1)	—^a^	.06
Age (years), mean (SD)	22.85 (1.28)	23.20 (1.94)	1.08 (103)	.29
Completed neurology rotation, n (%)	42 (91.3)	43 (72.9)	—	.02^b^
Pretest score (0‐13), mean (SD)	8.25 (1.63)	7.69 (1.79)	–1.65 (103)	.10
Posttest score (0‐13), mean (SD)	9.15 (1.42)	8.29 (1.95)	–2.61 (103)	.01^b^
Score improvement (pre-post), mean (SD)	0.90 (1.73)	0.60 (2.60)	–0.70 (103)	.48

a Not available.

b Significant at *P*<.05.

**Figure 3.**
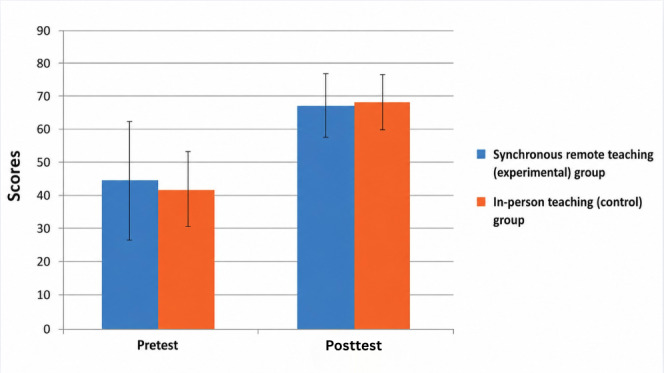
Comparison of pretest and posttest scores for the tendon reflex neurological examination objective structured clinical examination between the synchronous remote teaching (experimental) group and the in-person teaching (control) group.

Among the 105 participants who completed the knowledge test, 102 also completed the pretests and posttests for the tendon reflex neurological examination OSCE. The OSCE showed a moderate difficulty index of 0.55 and a good discrimination index of 0.43, indicating appropriate assessment performance for distinguishing learner ability. Within-group analyses showed that both the experimental group (n=46) and the control group (n=56) achieved substantial improvements in self-reported confidence and OSCE performance (all *P*<.001). These findings indicate that both instructional modalities were associated with marked short-term gains in psychomotor examination skills. However, regarding the primary outcome, the between-group difference in OSCE score improvement (pre-post change) was not statistically significant (mean 23.07, SD 16.53 vs mean 26.71, SD 11.31; *P*=.21), corresponding to a small effect size (Cohen *d*=−0.26; 95% CI −0.65 to 0.13). The CI included zero and spanned both potential benefits and disadvantages, indicating uncertainty in both the direction and magnitude of the effect. Overall, these findings suggest that the difference in OSCE-assessed psychomotor skill acquisition between the two teaching modalities was small and not educationally meaningful within the context of this study. Notably, a higher proportion of participants in the experimental group had prior neurology rotation experience (42/46, 91.3% vs 41/56, 73.2%; *P*=.02), which may have introduced baseline imbalance; however, this did not translate into a measurable advantage in OSCE improvement (Table 2 and Figure 3).

**Table 2.** Comparison of neurological tendon reflex objective structured clinical examination scores between the experimental and control groups.

Characteristic	Experimental group (n=46)	Control group (n=56)	*t* test (*df*)	*P* value	Cohen *D* (95% CI)
Female (%), n (%)	21 (45.7)	15 (26.8)	—^a^	.06	—
Age (years), mean (SD)	22.85 (1.28)	23.13 (1.82)	0.87 (100)	.39	—
Completed neurology rotation (%)	91.3 (42/46)	73.2 (41/56)	—	.02^b^	—
Frequency of practicing reflex examinations, mean (SD)	7.24 (14.93)	4.59 (13.16)	−0.95 (100)	.34	—
Preclass self-reported confidence in reflex examinations (1-5), mean (SD)	2.89 (0.95)	2.73 (1.12)	−0.77 (100)	.45	—
Postclass self-reported confidence in reflex examinations (1-5), mean (SD)	4.20 (0.78)	4.25 (0.75)	0.36 (100)	.72	—
Pretest total score (0‐80), mean (SD)	44.35 (17.83)	41.66 (11.18)	−0.89 (100)	.38	0.18 (−0.21 to 0.58)
Posttest total score (0‐80), mean (SD)	67.41 (9.47)	68.38 (8.31)	0.55 (100)	.59	−0.11 (−0.50 to 0.28)
Score Improvement (pre-post), mean (SD)	23.07 (16.53)	26.71 (11.31)	1.27 (100)	.21	−0.26 (−0.65 to 0.13)

a Not available.

b Significant at *P*<.05.

Cohen *d* was calculated for between-group differences using pooled SDs (experimental minus control); 95% CIs were calculated using the SE of *d*.

Further subgroup analyses based on specific examination sites and procedural steps did not show statistically significant between-group differences in pre- to posttest changes, as shown in Tables3  4. Importantly, the magnitude of changes across subcomponents was consistently small, and the direction of effects was not systematic, further supporting the absence of a meaningful modality-specific advantage.

**Table 3.** Subgroup analysis of tendon reflex objective structured clinical examination scores by examination site comparing the experimental and control groups.

Examination site and assessment	Experimental group (n=46), mean (SD)	Control group (n=56), mean (SD)	*t* test (*df*)	*P* value
Biceps brachii				
Pretest (0‐16)	9.13 (3.90)	8.79 (3.00)	−0.50 (100)	.62
Posttest (0‐16)	13.61 (1.89)	13.54 (2.08)	−0.18 (100)	.86
Improvement	4.48 (3.93)	4.75 (2.58)	0.40 (100)	.69
Brachioradialis				
Pretest (0‐16)	8.17 (4.72)	6.84 (3.95)	−1.56 (100)	.12
Posttest (0‐16)	13.48 (2.43)	13.54 (2.26)	0.12 (100)	.90
Improvement	5.30 (4.20)	6.70 (4.27)	1.65 (100)	.10
Triceps brachii				
Pretest (0‐16)	8.20 (4.18)	8.80 (2.50)	0.87 (100)	.39
Posttest (0‐16)	13.17 (2.89)	13.50 (2.56)	0.60 (100)	.55
Improvement	4.98 (4.47)	4.70 (3.35)	−0.35 (100)	.72
Knee jerk				
Pretest (0‐16)	10.33 (3.33)	9.52 (2.76)	−1.34 (100)	.18
Posttest (0‐16)	13.72 (2.18)	13.96 (1.80)	0.63 (100)	.53
Improvement	3.39 (3.69)	4.45 (2.78)	1.65 (100)	.10
Ankle jerk				
Pretest (0‐16)	8.52 (4.24)	7.71 (2.67)	−1.12 (100)	.27
Posttest (0‐16)	13.43 (2.46)	13.84 (2.12)	0.89 (100)	.37
Improvement	4.91 (4.06)	6.13 (3.02)	1.68 (100)	.10

**Table 4.** Subgroup analysis of tendon reflex objective structured clinical examination by examination step comparing the experimental and control groups.

Examination step and assessment	Experimental group (n=46), mean (SD)	Control group (n=56), mean (SD)	*t* test (*df*)	*P* value
Step 1: instruction				
Pretest (0‐10)	5.50 (3.21)	5.23 (3.06)	−0.43 (100)	.67
Posttest (0‐10)	6.78 (3.12)	7.04 (3.38)	0.39 (100)	.70
Improvement	1.28 (4.28)	1.80 (4.11)	0.63 (100)	.53
Step 2: preparation				
Pretest (0‐30)	17.41 (7.75)	16.29 (4.50)	−0.87 (100)	.39
Posttest (0‐30)	26.46 (5.25)	26.95 (3.39)	0.55 (100)	.59
Improvement	9.04 (7.29)	10.66 (5.15)	1.27 (100)	.21
Step 3: technique				
Pretest (0‐20)	10.98 (5.52)	10.52 (3.18)	−0.50 (100)	.62
Posttest (0‐20)	18.26 (2.28)	18.32 (2.26)	0.13 (100)	.89
Improvement	7.28 (5.26)	7.80 (3.34)	0.58 (100)	.56
Step 4: response				
Pretest (0‐20)	10.46 (5.20)	9.63 (4.67)	−0.85 (100)	.40
Posttest (0‐20)	15.91 (3.27)	16.07 (3.43)	0.24 (100)	.81
Improvement	5.46 (5.09)	6.45 (4.79)	1.01 (100)	.32

To further address potential baseline imbalances between groups, additional covariate-adjusted analyses were conducted for both knowledge and tendon reflex OSCE outcomes using an ANCOVA framework. Posttest outcomes were specified as dependent variables, with teaching modality as the fixed factor, and baseline performance and prior neurology rotation experience included as covariates.

For the knowledge assessment, covariate adjustment for baseline knowledge scores and prior neurology rotation experience did not materially alter the primary findings. The adjusted posttest knowledge score was numerically higher in the synchronous remote teaching group than in the in-person group (adjusted mean difference=0.94 points; 95% CI −0.10 to 1.98), corresponding to a small effect size (*F*(1,100)=3.23; *P*=.08; partial η²=0.031). Neither baseline knowledge performance nor prior neurology rotation experience demonstrated a significant independent association with post-test knowledge outcomes, and no significant interaction was observed between teaching modality and prior rotation experience.

Similarly, for the tendon reflex OSCE, covariate-adjusted analysis revealed a small, nonsignificant adjusted mean difference in posttest OSCE performance between the synchronous remote teaching and in-person teaching groups (−1.28 points; 95% CI −6.27 to 3.71), corresponding to a negligible effect size (*F*(1,98)=0.26; *P*=.61; partial η²=0.003). Baseline OSCE performance was a significant predictor of posttest OSCE scores (*P*<.001), whereas prior neurology rotation experience was not. No interaction effect between teaching modality and prior rotation experience was observed (*P*=.97), indicating that the effectiveness of synchronous remote teaching was consistent regardless of prior clinical exposure.

In view of the potential impact of prior neurology rotation experience on learning outcomes, additional analyses were conducted. Among the 105 participants, 85 had completed a neurology rotation, while 20 had not. The participants with prior rotations demonstrated slightly higher baseline knowledge scores; however, this difference was not statistically significant. No meaningful differences were observed in posttest knowledge scores or in pre-post score improvement between those with and without prior rotations (Table 5).

**Table 5.** Comparison of knowledge outcomes between participants with and without prior neurology rotations^a^.

Outcome measure	With prior neurology rotation (n=85), mean (SD)	Without prior neurology rotation (n=20), mean (SD)	*U* value	*z* value	*P* value
Pretest score (0‐13)	8.03 (1.68)	7.54 (1.98)	756.5	−0.76	.45
Posttest score (0‐13)	8.69 (1.82)	8.57 (1.62)	843.5	−0.05	.96
Score improvement (pre-post)	0.66 (2.11)	1.03 (2.85)	822.0	−0.23	.82

a Significant at *P*<.05. Subgroup analyses are exploratory. A formal interaction test (teaching modality × prior neurology rotation experience) was performed using a covariate-adjusted analysis of covariance framework; no significant interaction was observed.

Exploratory analyses suggested a numerically greater improvement in knowledge test scores among participants without prior neurology rotations, although this difference did not reach statistical significance. To formally evaluate whether prior neurology rotation experience moderated the effect of teaching modality on knowledge outcomes, we conducted a covariate-adjusted ANCOVA including baseline knowledge score, teaching modality, prior rotation experience, and their interaction. The interaction between teaching modality and prior neurology rotation experience was not statistically significant, indicating no evidence that prior clinical exposure moderated knowledge outcomes.

Among the 102 participants who completed the tendon reflex OSCE, those with prior neurology rotations (n=83) reported more frequent prior practice, higher precourse self-reported confidence, and higher pretest OSCE scores than those without rotations (n=19). Despite these baseline differences, postcourse self-reported confidence and posttest OSCE performance were similar between groups (Table 6). Exploratory subgroup analyses indicated a greater magnitude of OSCE score improvement among the participants without prior neurology rotations. However, this pattern should be interpreted cautiously, as the observed differences were small, derived from post hoc comparisons, and subject to potential type I error due to multiple testing.

**Table 6.** Comparison of tendon reflex objective structured clinical examination scores between participants with and without prior neurology rotations.

Outcome measure	With prior neurology rotation (n=83), mean (SD)	Without prior neurology rotation (n=19), mean (SD)	*U* value	*z* value	*P* value
Frequency of practicing reflex exams	6.53 (15.38)	2.53 (2.27)	508.0	−2.46	.01^a^
Preclass self-reported confidence in reflex exams (1-5)	2.90 (1.01)	2.37 (1.12)	557.5	−2.10	.04^a^
Postclass self-reported confidence in reflex exams (1-5)	4.23 (0.79)	4.21 (0.63)	744.0	−0.43	.67
Pretest total score (0‐80)	44.46 (14.91)	35.95 (10.58)	488.0	−2.58	.01^a^
Posttest total score (0‐80)	67.86 (8.70)	68.32 (9.59)	747.5	−0.35	.72
Score improvement (pre-post)	23.40 (14.17)	32.37 (10.43)	490.0	−2.57	.01^a^

a Significant at *P*<.05. Subgroup analyses are exploratory. A formal interaction test (teaching modality × prior neurology rotation experience) was performed using an analysis of covariance framework to evaluate effect modification (*P* for interaction=.97).

To assess whether prior neurology rotation experience moderated the effect of teaching modality on OSCE performance, a covariate-adjusted ANCOVA was performed with posttest OSCE score as the dependent variable, baseline OSCE score as a covariate, and teaching modality, prior rotation experience, and their interaction included as fixed factors. The interaction between teaching modality and prior neurology rotation experience was not statistically significant (*P* for interaction=.97), indicating that prior clinical exposure did not meaningfully influence the effect of teaching modality on psychomotor performance.

Subgroup analyses based on specific examination sites and procedural steps indicated that the participants with prior neurology rotations demonstrated higher baseline OSCE scores across several reflex sites and examination steps (Tables7  8). Postcourse improvements were numerically greater among the participants without prior rotations in several components. These site- and step-based analyses involved multiple comparisons and were conducted for exploratory purposes only; therefore, the reported *P* values were not adjusted for multiple testing and should be interpreted cautiously.

**Table 7.** Subgroup analysis of tendon reflex examination, objective structured clinical examination scores by examination site among participants with and without prior neurology rotations.

Examination site and assessment	With prior neurology rotation (n=83), mean (SD)	Without prior neurology rotation (n=19), mean (SD)	*U* value	*z* value	*P* value
Biceps brachii					
Pretest (0‐16)	9.30 (3.44)	7.37 (2.93)	485.0	−2.62	.009^a^
Posttest (0‐16)	13.66 (1.88)	13.16 (2.43)	715.0	−0.64	.52
Improvement	4.36 (3.30)	5.79 (2.76)	570.5	−1.89	.06
Brachioradialis					
Pretest (0‐16)	7.76 (4.51)	6.05 (3.29)	590.5	−1.71	.08
Posttest (0‐16)	13.48 (2.29)	13.63 (2.54)	717.5	−0.62	.54
Improvement	5.72 (4.40)	7.58 (3.39)	591.5	−1.70	.09
Triceps brachii					
Pretest (0‐16)	8.89 (3.46)	6.95 (2.37)	487.5	−2.61	.009^a^
Posttest (0‐16)	13.24 (2.85)	13.84 (1.92)	712.0	−0.67	.50
Improvement	4.35 (4.00)	6.89 (2.40)	432.5	−3.07	.002^a^
Knee jerk					
Pretest (0‐16)	10.06 (3.06)	9.11 (2.90)	666.0	−1.06	.29
Posttest (0‐16)	13.89 (1.78)	13.68 (2.71)	747.5	−0.36	.72
Improvement	3.83 (3.29)	4.58 (3.06)	674.0	−0.99	.32
Ankle jerk					
Pretest (0‐16)	8.45 (3.51)	6.47 (2.84)	490.5	−2.57	.01^b^
Posttest (0‐16)	13.58 (2.28)	14.00 (2.29)	669.0	−1.05	.30
Improvement	5.13 (3.53)	7.53 (3.08)	476.5	−2.69	.007^a^

a Significant at *P*<.01.

b Significant at *P*<.05.

**Table 8.** Subgroup analysis of tendon reflex examination objective structured clinical examination scores by examination step among participants with and without prior neurology rotations.

Examination step and assessment	With prior neurology rotation (n=83), mean (SD)	Without prior neurology rotation (n=19), mean (SD)	*U* value	*z* value	*P* value
Step 1: instruction					
Pretest (0‐10)	5.35 (3.03)	5.37 (3.55)	786.5	−0.02	.99
Posttest (0‐10)	6.81 (3.34)	7.42 (2.89)	720.5	−0.61	.54
Improvement	1.46 (4.20)	2.05 (4.12)	731.0	−0.50	.62
Step 2: preparation					
Pretest (0‐30)	17.46 (6.28)	13.89 (SD 4.84)	523.5	−2.28	.02^a^
Posttest (0‐30)	26.77 (SD 4.30)	26.53 (SD 4.46)	782.0	−0.06	.95
Improvement	9.31 (SD 6.07)	12.63 (SD 6.34)	571.5	−1.87	.06
Step 3: technique					
Pretest (0‐20)	11.18 (SD 4.52)	8.74 (SD 3.07)	474.0	−2.72	.007^b^
Posttest (0‐20)	18.23 (SD 2.29)	18.58 (SD 2.12)	745.5	−0.40	.69
Improvement	7.05 (SD 4.39)	9.84 (SD 3.06)	451.0	−2.92	.004^b^
Step 4: response					
Pretest (0‐20)	10.47 (SD 4.99)	7.95 (SD 4.03)	552.5	−2.03	.04^a^
Posttest (0‐20)	16.05 (SD 3.14)	15.79 (SD 4.21)	769.5	−0.17	.87
Improvement	5.58 (SD 4.97)	7.84 (SD 4.39)	575.0	−1.84	.07

a Significant at *P*<.05.

b Significant at *P*<.01.

All participants completed a pretest course evaluation survey assessing motivational beliefs regarding SRL (MSLQ) and technology acceptance of remote teaching products (TAM). Baseline scores were similar between the experimental group (n=46) and the control group (n=56) for both MSLQ (mean 90.17, SD 8.72 vs mean 89.79, SD 10.49; *P*=.84) and TAM (mean 60.54, SD 11.34 vs mean 59.75, SD 11.47; *P*=.73), indicating comparable baseline learner characteristics.

**Figure 4.**
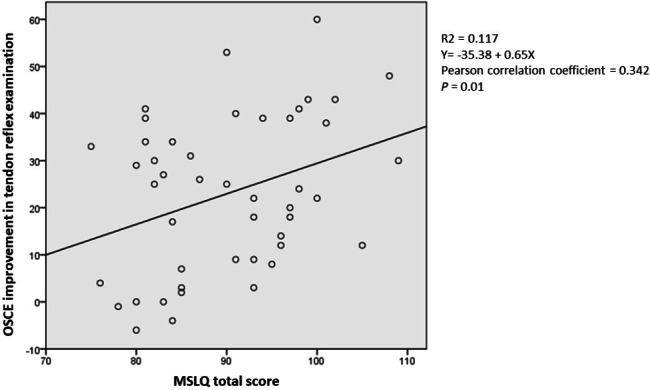
Linear regression curve between the Motivated Strategies for Learning Questionnaire (MSLQ) total score and improvement in objective structured clinical examination (OSCE) scores for tendon reflex examination in the experimental group.

To explore whether learning outcomes were associated with motivational beliefs, participants were stratified into high- and low-scoring groups based on the median MSLQ score. These analyses were conducted as exploratory and hypothesis-generating, and no formal adjustment for multiple comparisons was applied. Participants with higher motivational belief scores demonstrated higher posttest knowledge scores (mean 9.06, SD 1.77 vs mean 8.35, SD 1.71; *P*=.04) and greater OSCE improvement (mean 27.75, SD 13.72 vs mean 22.16, SD 13.76; *P*=.04) compared with those with lower scores. Within the experimental group, higher motivational scores were associated with higher post-test knowledge scores (mean 9.55, SD 1.42 vs mean 8.68, SD 1.30; *P*=.04) and greater OSCE improvement (mean 27.60, SD 15.77 vs mean 17.67, SD 16.13; *P*=.04). However, these subgroup findings remain exploratory and should not be interpreted as evidence of a causal relationship. Correlation analyses showed moderate positive associations between self-efficacy (*r*=0.367) and overall MSLQ score (*r*=0.342) with OSCE improvement, suggesting that higher motivational beliefs may be related to greater performance gains. However, the strength of these associations was moderate, and regression analysis indicated that motivational beliefs explained only a limited proportion of the variance in OSCE improvement (*R*^2^=0.117), as shown in Figure 4. These findings suggest that, while motivational factors may contribute to learning outcomes, they represent only one of multiple influencing factors.

We further examined whether learning outcomes differed according to levels of technology acceptance. Participants were stratified into high- and low-scoring groups based on the median TAM score. No significant differences were observed in knowledge improvement (mean 0.75, SD 2.02 vs mean 0.75, SD 2.49; *P*>.99) or OSCE performance (mean 24.87, SD 15.56 vs mean 25.28, SD 12.23; *P*=.88) between groups. These findings suggest that, within the context of this study, variation in technology acceptance was not associated with differences in short-term learning outcomes, although this may reflect a threshold effect whereby basic usability was sufficient for effective participation.

## Discussion

### Principal Findings

This study evaluated the short-term effectiveness of synchronous remote teaching compared with traditional in-person teaching for neurological examination training among fifth-year medical students. Both teaching modalities were associated with improvements in neurological examination knowledge, tendon reflex OSCE performance, and self-reported confidence. Although the synchronous remote teaching group demonstrated modestly higher postcourse knowledge scores, no statistically significant between-group differences were observed in OSCE improvement after covariate adjustment. Exploratory analyses further suggested that motivational beliefs related to SRL may be associated with greater psychomotor skill improvement in synchronous remote learning environments.

The absence of meaningful between-group differences in tendon reflex OSCE improvement suggests that synchronous remote teaching may support short-term acquisition of selected psychomotor neurological examination skills under structured instructional conditions. One possible explanation is that tendon reflex assessment relies on standardized procedural sequences, visual observation, and repeated guided practice, all of which can be effectively demonstrated through synchronous video-based interaction [10,36]. In addition, the RCPS system enabled close-up visualization of tendon localization, examination technique, and patient positioning, while instructors provided immediate corrective feedback during skill performance. These findings are consistent with prior literature suggesting that synchronous remote teaching environments may support psychomotor clinical skills when real-time interaction and structured feedback are incorporated [17,36,37]. However, unlike many previous studies that relied primarily on self-reported outcomes or asynchronous formats, this study evaluated performance using an OSCE-based assessment focused on a specific neurological examination task.

As this study used a nonrandomized design, baseline imbalance in prior neurology rotation experience required careful interpretation. Participants with prior rotations demonstrated higher baseline self-reported confidence and greater OSCE performance, suggesting that prior clinical exposure may contribute to foundational procedural skills [38]. However, covariate-adjusted analyses and formal interaction testing did not demonstrate evidence that prior neurology rotation experience modified the effect of teaching modality on either knowledge or OSCE outcomes. These findings suggest that the observed learning outcomes associated with synchronous remote teaching appeared consistent across different baseline experience levels, although residual confounding cannot be fully excluded.

### Theoretical Implications: Interpreting Findings Through the Lenses of SRL Theory and TAM

The exploratory association between motivational beliefs and OSCE improvement may be interpreted through the lens of SRL theory. In particular, self-efficacy and motivational regulation may support learners’ engagement, persistence, and responsiveness to feedback during psychomotor skill acquisition [39-41]. These associations appeared more pronounced in the synchronous remote teaching group, suggesting that remote interactive environments may place greater emphasis on learners’ active self-monitoring and self-regulatory capacity [38,42]. However, because these analyses were exploratory and this study was not designed to directly evaluate SRL mechanisms, these findings should be interpreted cautiously and regarded as hypothesis-generating.

In contrast, technology acceptance was not significantly associated with knowledge or OSCE outcomes. One possible interpretation is that, within highly interactive and instructor-guided synchronous environments, technology acceptance may function primarily as a prerequisite for participation rather than a direct determinant of learning effectiveness [21,43]. This distinction may help contextualize the role of TAM in synchronous psychomotor clinical skills training, although the present findings remain exploratory and do not establish theoretical mechanisms.

Collectively, these findings suggest that the effectiveness of synchronous remote psychomotor skills training may depend less on technology acceptance itself and more on how learners actively engage with feedback and regulate their own learning processes. From a theoretical perspective, this observation is broadly consistent with SRL models, which emphasize self-monitoring, self-efficacy, and strategic regulation as important determinants of learning performance in complex educational environments [22,23,42]. In contrast, TAM may be more useful for explaining learners’ willingness to adopt and engage with educational technologies than for directly predicting skill acquisition outcomes once active participation has been established [19-21]. Although causal mechanisms cannot be inferred from this study, these findings contribute to the growing literature exploring how learner characteristics interact with technology-mediated clinical skills education and may help inform future theory-driven investigations of remote psychomotor skills training.

### Practical and Real-World Implications

From a practical perspective, synchronous remote teaching may provide a flexible option for delivering neurological examination training when face-to-face instruction is constrained by geographical distance, scheduling limitations, infection control requirements, or limited access to specialist instructors. Enhanced telepresence systems and synchronous video-based educational environments may facilitate real-time demonstration, observation, and feedback while expanding access to clinical skills education across distributed training sites [17,29,36]. Although further evidence is required regarding scalability and long-term effectiveness, the present findings suggest that structured synchronous remote teaching may serve as a useful supplementary instructional approach for medical schools seeking to maintain interactive clinical skills training while addressing logistical challenges in contemporary medical education [36,37].

### Limitations

Several limitations should be considered when interpreting the present findings. First, this study used a quasi-experimental design without randomization, which introduces potential selection bias and residual confounding. In particular, baseline imbalance in prior neurology rotation experience may have influenced learning outcomes despite the use of covariate-adjusted analyses and formal interaction testing. Therefore, the findings should be interpreted as associative rather than strictly causal. In addition, participants’ awareness of being studied may have introduced a Hawthorne effect [44], potentially increasing motivation and engagement across both groups. Several subgroup and exploratory analyses were conducted without formal correction for multiple testing, which increases the potential risk of type I error; accordingly, these exploratory findings should be regarded as hypothesis-generating rather than confirmatory. Furthermore, self-reported confidence represents a subjective learner perception and may not fully correspond to objectively measured psychomotor performance.

Second, the study evaluated only immediate posttest outcomes and, therefore, does not establish long-term retention or durable skill acquisition. In addition, the directly observed psychomotor assessment focused specifically on tendon reflex examination skills, which represent only one component of the neurological examination; therefore, the findings should not be generalized to broader neurological examination competencies without further study. The pragmatic design also did not include a formal a priori sample size calculation, and unequal group sizes resulted from cohort availability and scheduling constraints, which may have limited statistical power to detect small between-group differences. Finally, the synchronous remote teaching sessions were conducted in relatively small groups with substantial instructor interaction and individualized feedback; therefore, the scalability and effectiveness of this instructional approach in larger educational settings remain uncertain.

### Conclusions

Under the conditions of this quasi-experimental study, synchronous remote teaching was associated with short-term improvements in tendon reflex examination skills, neurological examination–related knowledge, and self-reported confidence among fifth-year medical students. Although modestly higher postcourse knowledge scores were observed in the synchronous remote teaching group, no statistically significant between-group differences were identified in OSCE-assessed psychomotor performance after covariate adjustment. These findings suggest that structured synchronous remote teaching may support short-term acquisition of selected neurological examination skills when real-time interaction, guided practice, and immediate feedback are incorporated into instructional design.

To our knowledge, this study is among the first to evaluate synchronous remote teaching for tendon reflex examination training using OSCE-based psychomotor assessment and theory-informed interpretation grounded in SRL and technology acceptance frameworks. These findings contribute to the growing literature on synchronous digital clinical skills education by extending prior research that has primarily focused on asynchronous learning or subjective learner perceptions. However, because this study assessed only immediate outcomes and focused on tendon reflex examination skills, the findings should not be interpreted as evidence of long-term skill retention or generalized effectiveness across the full spectrum of neurological examination competencies. Future research should incorporate randomized designs, larger multicenter cohorts, and longitudinal follow-up to evaluate scalability, long-term retention, transferability to other neurological examination domains, and strategies to support SRL in remote clinical skills education.

## Abbreviations

ANCOVA: analysis of covariance
MSLQ: Motivated Strategies for Learning Questionnaire
OSCE: objective structured clinical examination
RCPS: Remote Clinical Presence System
SRL: self-regulated learning
TAM: technology acceptance model
